# Devergie’s Ointment for Chilblains

**Published:** 1846-06

**Authors:** 


*Devergie's Ointment for Chilblains.*—Lard, seven drachms and a half; creasote, ten drops; solution subacetate of lead, ten drops; watery extract of opium, a grain. Mix for an ointment.—*Dub. Hos. Gaz.—Ibid.*

